# Identifying risks for severity of neurological symptoms in Hungarian West Nile virus patients

**DOI:** 10.1186/s12879-020-05760-7

**Published:** 2021-01-13

**Authors:** Márton Koch, Éva Pozsgai, Viktor Soós, Anna Nagy, János Girán, Norbert Nyisztor, Tibor Martyin, Zsófia Müller, Melánia Fehér, Edit Hajdú, Csaba Varga

**Affiliations:** 1 Department of Emergency Medicine, Somogy County Kaposi Mór Teaching Hospital, Tallián Gyula Street, 20-32, Kaposvár, 7400 Hungary; 2 Department of Public Health, Medical School, University of Pécs, Szigeti Street, 12, Pécs, 7624 Hungary; 3 Institute of Primary Health Care, Medical School, University of Pécs, Rákóczi Street 2, Pécs, 7623 Hungary; 4 National Reference Laboratory for Viral Zoonoses; National Public Health Center, 1097 Albert Flórián Road 2-6, Budapest, Hungary; 5 Department of Infectious Diseases (Hepatology and Immunology), Békés County Central Hospital, Semmelweis Street 1, Gyula, 5700 Hungary; 6 Department of Infectious Diseases, Fejér County St George Teaching Hospital, Seregélyesi Street 3, Székesfehérvár, 8000 Hungary; 7 Department of Infectology, University of Szeged, Albert Szent-Györgyi Health Center, Kálvária Avenue 57, Szeged, 6725 Hungary; 8 Institute of Emergency Care and Pedagogy of Health, Faculty of Health Sciences, University of Pécs, Vörösmarty Mihály Street 4, Pécs, 7621 Hungary

**Keywords:** West Nile virus infection, West Nile neuroinvasive disease, Neurological outcome, Modified Rankin scale, Emergency department

## Abstract

**Background:**

West Nile virus (WNV) infections have become increasingly prevalent in certain European countries, including Hungary. Although most human infections do not cause severe symptoms, in approximately 1% of cases WNV infections can lead to severe WNV neuroinvasive disease (WNND) and death. The goal of our study was to assess the neurological status changes of WNV –infected patients admitted to inpatient care and to identify potential risk factors as underlying reasons for severe neurological outcome.

**Methods:**

We conducted a retrospective chart review of 66 WNV-infected patients from four Hungarian medical centers. Patients’ neurological status at hospital admission and at two follow-up intervals (1st follow-up, within 60–90 days and 2nd follow-up, within 150–180 days, after hospital discharge) were assessed. All of the 66 patients in the initial sample had some type of neurological symptoms and 56 patients were diagnosed with WNND. The modified Rankin Scale (mRS) and the West Nile Virus Neurological Index (WNV-N Index), a scoring system designed for the purpose of this study, were used for neurological status assessment. Patients were dichotomized into two categories, “moderately severe” and “severe” based on their neurological status. Descriptive analysis for sample description, stratified analysis for calculation of odds ratio (OR) and logistic regression for continuous input variables, were performed.

**Results:**

The average number of days between the onset of neurological symptoms and hospital admission (the neurological symptom interval) was 6.01 days. Complications during the hospital stay arose in almost a fifth of the patients (18.2%) and 5 patients died. Each day’s increase in the neurological symptom interval significantly increased the risk for developing a severe neurological status following hospital admission (0.799-fold and 0.688-fold, based on the WNV-N Index and mRS, respectively). Patients’ age, comorbidity, presence of complications and symptoms of malaise, and gait uncertainty were shown to be independent risk factors for severe neurological status.

**Conclusions:**

Timely hospital admission of patients with neurological symptoms as well as risk assessment by clinicians - possibly with an optimal assessment tool for estimating neurological status- could improve the neurological outcome of WNV-infected patients.

## Background

West Nile virus (WNV) has become an endemic source of disease in a number of countries worldwide [1]*.* Although most human infections are asymptomatic or can be characterized as a mild disease, in rare cases WNV infections can lead to severe neuroinvasive disease and death [2]. Increasing trends in WNV infections have been reported in certain European countries, including Hungary, between 2014 and 2018 [3]. Compared to the average number of cases in preceding years, there was a 10-fold increase in 2018, with the Central and Eastern counties of Hungary being mostly affected [4, 5].

The WNV is a flavivirus and it is transmitted to humans by mosquito-bites, thus, causing a zoonotic disease [6]*.* Approximately 80% of patients infected by WNV remain asymptomatic and about 20% develop West Nile Fever, a mild, flu-like disease [2]. 1% of all infections results in West Nile neuroinvasive disease (WNND), which may manifest itself as meningitis, encephalitis, meningoencephalitis or acute flaccid paresis (AFP) [7]. WNND is a serious disease, with a mortality rate between 10 and 30%, and often leads to different forms of disability or deficit, even after recovery from the acute illness [2].

Studies investigating long-term prognosis after WNND, have shown that full neurological recovery could be as low as 37% after 12 months, and most patients (86%) who had suffered from encephalitis still had abnormal neurological exam findings up to 1–3 years later [8, 9].

Assessing risks for disease outcome is important for clinicians, particularly in WNND, where lack of timely or adequate supportive therapy may lead to lingering neurological symptoms and even long-term disabilities. Age, male gender and ongoing chronic illnesses, such as hypertension as risk factors, have been shown to be associated with developing WNND, while age, congestive heart failure, chronic hepatitis and neoplasm have been found to be risk factors for death [10, 11]. Investigations taking into account various parameters related to the patients and aimed at identifying potentially novel risk factors greatly enhance knowledge related to optimal medical care for WNND patients.

The goal of our study was to analyze the neurological status of West Nile Virus –infected patients upon hospital admission and to assess changes in their status after a short-term follow-up period, using one established and one novel neurological assessment tool. By collecting data regarding patients’ demographic, symptomatic, diagnostic and treatment-related characteristics, it was also our objective to identify potential risk factors as underlying reasons for severe neurological outcome.

## Methods

### Study design

The authors obtained ethical approval to conduct the investigation. A retrospective chart review was carried out on patients with a laboratory-confirmed diagnosis of West Nile virus disease admitted to four Hungarian medical centers (Somogy County Kaposi Mór Teaching Hospital, Békés County Central Hospital, the Albert Szent-Györgyi Healthcare Centre of the University of Szeged and the Fejér County St George Teaching Hospital) found in counties afflicted moderately or highly with West Nile fever or WNND (based on data from 2018) [12]. Patients aged > 18 years old, admitted to any of the four previously indicated Hungarian regional medical centers within a 5-year study period (31.12.2014–01.01.2020.) with laboratory-confirmed West Nile virus infection were included in the study. A total of 66 patients were identified in our study.

### WNV laboratory testing

WNV infections are defined as laboratory confirmed or probable cases according to the European Union’s case definition criteria 2012/506/EU [13]. For laboratory case confirmation at least one of the following four criteria must be met: 1. Isolation of the virus from blood or cerebrospinal fluid (CSF); 2. detection of WNV nucleic acid in blood or CSF; 3. detection of WNV specific IgM antibodies in CSF; 4. detection of anti-WNV IgM antibodies in high titre and detection of anti-WNV IgG antibodies, and confirmation by neutralization. The presence of WNV specific antibodies in a serum sample allows probable case classification.

The laboratory diagnosis of acute human WNV cases included both serological and molecular biological methods, performed only at the National Reference Laboratory for Viral Zoonoses (National Public Health Center; Budapest, Hungary).

For detection of virus-specific antibody response, including IgG, IgM and IgA determination, *in house* indirect-immunofluorescent assay and capture IgM ELISA test (Enzyme-Linked Immunosorbent Assay; Focus Diagnostics, DiaSorin Molecular LLC, Cypress, CA, USA) were used. Testing for other endemic flaviviruses, such as *Tick-borne encephalitis virus* (TBEV) and *Usutu virus* (USUV) were also an integral part of the differential diagnostic algorithm, as serological-cross reactivity is the major challenge of laboratory assays based on flavivirus-antibody determination [14–17].

For confirmation WNV microneutralization-assay was carried out, according to a protocol described elsewhere with minor modifications [18].

Serum and CSF specimens were collected for serological diagnostic tests, while in cases where anticoagulant-treated whole blood and/or urine samples were available, viral nucleic acid amplification tests were also performed. A real-time reverse-transcription polymerase chain reaction (RT-PCR) targeting a conservative, untranslated region of the viral genome allows the rapid detection of lineage 1 and lineage 2 WNV RNA. For verification a nested RT-PCR method, comprising other sets of oligonucleotides was used, followed by amplicon-based bidirectional Sanger sequencing. Sequences were regularly submitted to the NCBI (National Center for Biotechnology Information) GenBank database [19].

### Data collection and analysis

Data from the medical records was collected from the databases of the four medical centers by the study investigators.

Collected data included (1.) demographic characteristics: age, gender (2.) medical history: ongoing illnesses and relevant medications (3.) clinical features: presenting general and neurological symptoms, onset of neurological symptoms (4.) diagnostic and therapeutic interventions (5.) complications and length of hospital stay (5.) neurological follow-up data.

Information regarding ongoing illnesses (chronic kidney disease, liver disease, hypertension, chronic alcoholism, asthma, chronic obstructive pulmonary disease, diabetes mellitus, heart disease, cancer, autoimmune and neurological diseases) as well as relevant (immunosuppressive) medication were gathered.

We determined the number of days between the onset of neurological symptoms and hospital admission.

Presenting symptoms included all general and neurological symptoms which patients had at hospital admission and/or at the first detailed physical (including neurological) examination within 24 h of admission.

Data regarding neurological symptoms and status were assessed in detail at both presentation and follow-up, based on the principles of the Neuropathy Impairment Score (NIS) [20] (which was published as the revision of the Neuropathy Disability Score (NDS) [21]), but with broader categories for the purpose of our study: muscle strength, reflexes and sensory alterations were evaluated.

Meningitis was diagnosed if cerebrospinal fluid (CSF) pleocytosis or acute infection (confirmed by PCR, culture or serology) alone or combined with clinical evidence of meningeal inflammation or acute infection, fever, hypothermia, or neuroimaging findings consistent with acute meningeal inflammation were present. Encephalitis was defined as altered level of consciousness and at least two of the following: additional evidence of central nervous system inflammation plus neuroimaging findings consistent with acute inflammation or acute demyelination, presence of focal neurologic deficit, electroencephalography findings consistent with encephalitis, generalized or partial seizures [7, 22]. When criteria for both meningitis and encephalitis were met, the cases were classified as meningoencephalitis. The definition of acute flaccid paresis included acute onset of limb weakness and at least two other findings: asymmetric to weakness, areflexia/hyporeflexia of affected limb(s), absence of pain, paresthesia, or numbness in affected limb(s), CSF pleocytosis and elevated protein levels, electrodiagnostic studies consistent showing anterior horn cell process or spinal cord magnetic resonance imaging documenting abnormal increased signal in the anterior gray matter [7, 22].

In order to facilitate the evaluation of patients’ neurological status upon admission and at follow-up, two assessment scales were used. One assessment scale, the modified Rankin scale (mRS) [23, 24] has been widely-used for assessing patients’ functional status after stroke and meningitis in previous studies. The scoring system of the mRS is shown in Table 1.The other assessment scale, called the West Nile Virus Neurological Index (WNV-N Index) was developed by our research team specifically for the purpose of this study. Points were assigned for each neurological symptom often present in a WNV infection affecting the Central Nervous System. Based on the number and degree of their neurological symptoms, each patient received a WNV-N Index value (at both admission and at follow-up, where possible). Patients were given “0” points for not having a certain neurological symptom, or if the examined neurological parameter was normal. The maximal total number of points, that could be given, was 25.5 points. The scoring system for the mRS has been described previously [23]. The scoring system for the WNV-N Index is shown in Table 2.

**Table 1:** Modified Rankin Scale (mRS) [25]

Score	Description
0	No symptoms
1	No significant disability despite symptoms; able to carry out all usual duties and activities
2	Slight disability: unable to carry out all previous activities, but able to look after own affairs without assistance
3	Moderate disability: requiring some help, but able to walk without assistance
4	Moderately severe disability; unable to walk and attend to bodily needs without assistance
5	Severe disability; bedridden, incontinent and requiring constant nursing care and attention
6	Dead

**Table 2:** Scoring system for the WNV-N Index

Neurological symptom		assigned points
Localisation of muscle weakness	normal	0
	lower body	0.5
	upper body	0.25
	lower body +upper body	1
Degree of muscle weakness^a^	normal	0
	25% weak	1
	50% weak	2
	75% weak	3
	can just move against gravity	3.25
	can just move with gravity eliminated	3.5
	muscle contraction can be felt or seen but no visible movement	3.75
	paralysis	4
Reflex involvement (decreased/absent)	yes	1
	no	0
Sensory involvement (decreased/absent)	yes	1
	no	0
Dizziness	yes	1
	no	0
Gait uncertainty	yes	0.5
	no	0
Tremor	yes	0.5
	no	0
Photophobia	yes	0.5
	no	0
Speech impairment	yes	0.5
	no	0
Slow speech	yes	0.5
	no	0
Confusion, altered mind state	yes	0.5
	no	0
Diplopia	yes	0.5
	no	0
Headache	yes	0.5
	no	0

^a^ Based on the principles of the Neuropathy Impairment Score [20]

Patients were dichotomized into two categories, according to their mRS and WNV-N Index scores.

Based on their mRS values, patients were considered to have (1.) **moderately severe neurological status** with a score below 3 and (2.) **severe neurological status** with a score of 3 or above.

Based on their WNV-N Index, patients were considered to have (1.) **moderately severe neurological status** with a score below 2.25 and (2.) **severe neurological status** with a score of 2.25 or above.

Data regarding patients’ neurological status at follow-up examinations were gathered within two intervals, 60–90 days, which we called the “1st follow-up” and within 150–180 days which we called the “2nd follow-up”, of discharge from the hospital. Due to the low number of patients at 2nd follow-up, comparisons and risk calculations were made for data from the 1st follow-up only.

### Statistical analysis

Statistical analysis was undertaken using IBM SPSS Statistics for Windows version 25. Descriptive analysis (Frequency and Crosstab) was carried out to describe the study sample. Comparison of the presenting neurological status with the follow-up status, characterized by the mRS and WNV-N Index, were performed using the Wilcoxon Signed Ranks Test. The risks for illness severity, regarding the dichotomized input variables were analyzed. To calculate the crude odds ratio (OR) for each risk as well as to determine the confounders and effect modifiers, stratified analysis was performed. For continuous input variables, logistic regression was used to calculate the crude OR. *P* < 0.05 was considered statistically significant.

## Results

### The demographic and general clinical characteristics of WNV patients

**Fig. 1:**
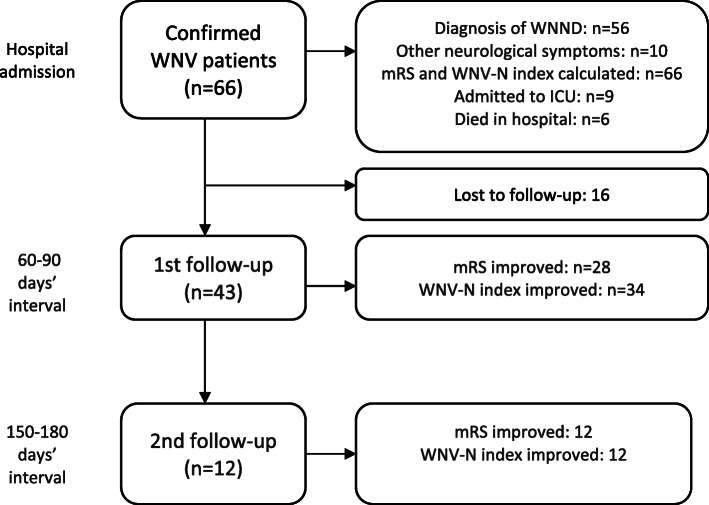
Distribution of patients and process of investigation at hospital admission and follow-up

A total of 66 patients with confirmed WNV infection were identified from four medical centers in Hungary within the five-year study period. Figure 1 shows the distribution of patients and process of investigation at hospital admission and follow-up.

The majority of the patients (63.3%) were male, and the average age of the patients was 56.74 years. Two-thirds of the patients (66.66%) was below 65 years, with almost a third of all patients being under the age of 45. Thirty-seven patients had none or only 1 ongoing illness, while 29 patients had at least two or more comorbidities at the time of admission to the hospital. Immunosuppressive medication (immunsuppressive, antitumor or steroid therapy) was taken regularly by 6 of the patients (9.1%). Following the highly prevalent “fever” (92.42%), most common general symptoms upon admission were “weakness/malaise” and “nausea/vomiting” (both 39.4%), while abdominal pain and upper respiratory tract symptoms like coughing, were the least frequent symptoms (both 7.6%). The average number of days between the onset of neurological symptoms and hospital admission was 6.01 days (Table 3).

**Table 3:** Demographic and general clinical characteristics of WNV patients (*n* = 66)

Gender and age distribution n (%)	
Male	42 (63.3)
Female	24 (36.4)
Total	66
Age (average)	56.74 years
Age (median)	60.0 years
**patients below 65 years old**	**43 (64.15)**
- patients < 45 years old	19 (28.79)
- patients 45-65 years old	24 (36.4)
**patients over 65 years old**	**23 (34.8)**
**Time between onset of neurological symptoms and hospital admission (days)**	
average	6.01
median	4.00
**Number of Comorbidities n (%)**	
None or 1 illness	37 (56.1)
2 or more illnesses	29 (43.9)
**Relevant medication n (%)**	
None	59 (89.3)
Immunsuppressive therapy	4(6.1)
Active antitumor therapy	1 (1.5)
Steroid therapy	1 (1.5)
Data not available (NA)	1 (1.5)
**General symptoms at presentation n (%)**	
Fever	61 (92.42)
Weakness and malaise	26 (39.4)
Nausea, vomiting	26 (39.4)
Skin rash	20 (30.3)
Diarrhea	7 (10.6)
Muscle/joint pain	7 (10.6)
Abdominal pain	5 (7.6)
Coughing, upper respiratory tract symptom	5 (7.6)

### Diagnostics, therapeutical interventions, and clinical outcomes of WNV patients

Cerebrospinal fluid analysis was performed in most (72.7%) of the cases. IgA, IgG and IgM were positive in both the serum and CSF in the vast majority of the patients (93.9%). PCR (Polymerase chain reaction) analysis showed positive results in a quarter of the patients (25.8%). All of the patients who tested positively for PCR had lineage 2 WNV infection. (Table 4A) Regarding treatment, almost half of the patients (48.5%) received both antiviral treatment (acyclovir for suspected herpes simplex virus infection) and antibiotic therapy. The majority (65.1%) received some form of pain medication (59.1% minor, 4.5% major, 1.5% both minor and major analgesics) and 9 patients (13.6%) required intensive therapy. The combination of mannitol and steroid and mannitol alone were the most frequently administered forms of additional medication, given in 13 (19.7%) and 9 cases (13.64%), respectively. (Table 4B) Complications during the hospital stay arose in almost fifth of the patients (18.2%) and 5 patients died. Complications included respiratory failure, hearing loss, seizures, pneumonia and polyneuropathy. The average length of the hospital stay was 10.61 days, and the median length was 9 days. (Table 4C).

**Table 4:** Diagnostics (A), therapeutical interventions (B) and clinical outcomes (C) of WNV-infected patients

**(A) Diagnostics: WNV Laboratory testing, n (%)**		
Cerebrospinal fluid	yes	48 (72.7)
	no	8 (12.1)
	data unavailable	10 (15.2)
Serology	serum and CSF positive (IgG, A, M)	62 (93.9)
	serum positive (IgG, M)	3 (4.5)
	serum positive (IgG: inconclusive, IgA: borderline, IgM: positive)	1 (1.5)
PCR	positive	17 (5.8)
	negative	37 (55.9)
	data unavailable	10 (15.2)
	inconclusive	2 (3.0)
Lineage	lineage 2	17 (25.8)
	unknown (due to PCR-negative or inconclusive results)	39 (59,0)
	not applicable	10 (15.2)
**Other (relevant) Diagnostic procedures, n (%)**		
Imaging	Confirmed alteration with CT	10 (15.2)
	Confirmed alteration with MRI	7 (10.6)
	Confirmed alteration with both CT and MRI	19 (28.8)
	none	24 (36.4)
	data unavailable	6 (9.1)
**(B) Therapy, n (%)**		
Antibiotic/Antiviral treatment	Antibiotic treatment	12 (18.2)
	Antiviral treatment	1 (1.5)
	Antibiotic+ Antiviral treatment	32 (48.5)
	none	21 (31.8)
Pain medication	Minor analgesics	39 (59.1)
	Major analgesics	3 (4.5)
	Both minor and major analgesics	1 (1.5)
	none	21 (31.8)
	data unavailable	2 (3.0)
Other relevant medication	Steroid only	3 (4.55)
	Mannitol only	9 (13.64)
	Steroid + Mannitol	13 (19.70)
	Antiepileptics only	3 (4.55)
	Steroid + Mannitol + Sedative	5 (7.58)
	Steroid + Mannitol + medication for Dizziness	1 (1.52)
	Steroid + Mannitol + Sedative + Antiepileptics	1 (1.52)
	none	28 (42.42)
Intensive Care		9 (13.6)
**(C) Clinical outcome, n (%)**		
Complications (respiratory failure, hearing loss, seizures, pneumonia polyneuropathy)		7 (10.6)
Death		5 (7.6)
Length of hospital stay (days)	average	10.6
	median	9.0

### Neurological symptoms and status upon hospital admission, and at 1st and 2nd follow-up

All of the 66 patients’ neurological examinations and assessments were carried out within 24 h after hospital admission. Forty-three patients were followed up within 60–90 days, and 12 patients within 150–180 days after hospital discharge, which were called the 1st and 2nd follow-up appointments, respectively. (Table 3*;* Fig. 1).

Sixty patients had a Glasgow Coma Scale (GCS) value between 13 and 15, while one patient’s GCS value was below 8. Meningitis was confirmed in 8, encephalitis in 1, meningoencephalitis in 38 and acute flaccid paresis in 9 patients following hospital admission. Fifty-six patients were diagnosed with WNND, but all of the 66 patients had some type of neurological symptom: 19 patients (28.8%) suffered from muscle weakness, from which 10 had muscle weakness in both their upper and lower parts of their bodies, however, the degree of weakness in most of these cases was mild (NIS grade 1). Reflex and sensory involvement were present in 5 and 3 cases, respectively. The most common other neurological symptoms were headache, vertigo and confusion following hospital admission, 69.7, 45.5 and 27.3%, respectively. (Table 4).

None of the patients had meningitis, encephalitis, meningoencephalitis at their 1st and/or 2nd follow-up. Four patients (9.3%) had acute flaccid paresis at 1st follow-up but none were recorded to have it, subsequently. Seven patients (16.3%) suffered from muscle weakness and their degree of muscle weakness was moderate or severe (NIS grades 2–3.25). The number of patients with reflex and sensory involvement stayed almost unchanged compared to hospital admission (5 and 2 cases, respectively). Other neurological symptoms were comparatively rare. At 2nd follow-up, only one patient was found to have muscle weakness as well as reflex and sensory involvements. Other neurological symptoms were not found.

Based on their mRS categories, 27.3% of the patients’ neurological status was considered severe following hospital admission. At 1st follow-up only 14.0% were severe, and at 2nd follow-up all the patients were in the moderately severe category.

According to our WNV-N Index, 33.3% of the patients had a severe neurological status following hospital admission, which decreased to 14 and 8.3%, by the 1st and 2nd follow-up intervals, respectively. (Table 5).

**Table 5:** Neurological symptoms, status and assessment scales at presentation, 1st and 2nd follow-up

Neurological status, n (%)				
		**At hospital admission** **(*n*** **= 66)**	**1st Follow-up (60–90 days after discharge)** **(*n*** **= 43)**	**2nd Follow-up (150–180 days after discharge)** **(*n*** **= 12)**
GCS	13–15	62 (93.9)	–	–
	9–12	3 (4.5)	–	–
	8 or less	1 (1.5)	–	–
Meningitis	yes	8 (12.1)	0 (0)	0 (0)
	no	57 (86.4)	43 (100)	12 (100)
	data unavailable	1 (1.5)	0 (0)	0 (0)
Encephalitis	yes	1 (1.5)	0 (0)	0 (0)
	no	64 (97.1)	43 (100)	12 (100)
	data unavailable	1 (1.5)	0 (0)	0 (0)
Meningoencephalitis	yes	38 (40.9)	0 (0)	0 (0)
	no	27 (57.6)	43 (100)	12 (100)
	data unavailable	1 (1.5)	0 (0)	0 (0)
Acute flaccid paresis	yes	9 (13.6)	4 (9.3)	0 (0)
	no	57 (86.4)	39 (90.7)	12 (100)
Localisation of muscle weakness	none	46 (69.7)	36 (83.7)	11 (91.7)
	lower body	6 (9.1)	3 (7.0)	1 (8.3)
	upper body	3 (4.5)	1 (2.3)	0 (0)
	lower body + upper body	10 (15.2)	3 (7.0)	0 (0)
	unconscious	1 (1.5)	–	–
Degree of muscle weakness	0 (normal)	46 (69.7)	36 (83.7)	11 (91.7)
	1	11 (16.7)	2 (4.7)	0 (0)
	2	3 (4.5)	2 (4.7)	1 (8.3)
	3.25	4 (6.1)	2 (4.7)	0 (0)
	4	1 (1.5)	1 (2.3)	0 (0)
	unconscious	1 (1.5)	–	–
Reflex involvement (decreased or absent)	no	59 (89.4)	38 (88.4)	11 (91.7)
	yes	5 (7.6)	5 (11.6)	1 (8.3)
	unconscious	1 (1.5)	–	–
	data unavailable	1 (1.5)	–	–
Sensory involvement (decreased or absent)	no	61 (92.4)	41 (95.3)	11 (91.7)
	yes	3 (4.5)	2 (4.7)	1 (8.3)
	unconscious	1 (1.5)	–	–
	data unavailable	1 (1.5)	–	–
**Other Neurological symptoms**		**At hospital admission** **(*n*** **= 66)**	**1st Follow-up** **(*n*** **= 43)**	**2nd Follow-up** **(*n*** **= 12)**
Headache		46 (69.7)	5 (11.6)	–
Dizziness		30 (45.5)	7 (16.2)	–
Confusion, altered mind status		18 (27.3)	–	–
Tremor		7 (10.6)	–	–
Diplopia		4 (6.1)	–	–
Gait uncertainty		3 (4.5)	2 (4.65)	–
Speech disturbance		4 (6.0)	–	–
Photophobia		3 (4.5)	–	–
**Assessment tools, n (%)**		**At hospital admission**	**1st follow-up**	**2nd follow-up**
WNV-N Index	moderately severe	44 (66.7)	37 (86.0)	11 (91.7)
	severe	22 (33.3)	6 (14.0)	1 (8.3)
Modified Rankin Scale	moderately severe	48 (72.7)	37 (86.0)	12 (100)
	severe	18 (27.3)	6 (14.0)	0 (0)

### Changes in mRS and WNV-N index scores from presentation to 1st follow-up

We compared the status changes of patients until the 1st follow-up using the Wilcoxon test. Twenty patients’ mRS score improved, 13 patients’ score remained unchanged and only two patients’ mRS score worsened until the 1st follow-up. A similar tendency could be observed when comparing the WNV-N Index scores: 34 patients showed improvement, 7 remained unchanged and 2 patients status deteriorated. (Table 6).

**Table 6:** Changes in WNV-N index score and mRS score from presentation to 1st follow-up

Relation	Ranks	N
WNV-N index score at presentation and at 1st follow-up (Z = − 4.50; *p* ≤ 0.000)	Negative ranks	34
	Positive ranks	2
	Ties	7
	Total	43
mRS score at presentation and at 1st follow-up (Z = − 4.23; p ≤ 0.000)	Negative ranks	28
	Positive ranks	2
	Ties	13
	Total	43

### Risk factors for having a severe neurological status following hospital admission and at 1st follow-up

We investigated whether risk factors for worse (severe) neurological status upon hospital admission and at 1st follow-up could be identified. Therefore, the associations between demographic characteristics, the time interval between onset of neurological symptoms and hospital admission, clinical features, general symptoms and severe neurological status upon admission, based on the mRS and WNV-N Index, were analyzed. Also, relationships between diagnostic and therapeutic interventions, occurrence of complications and severe neurological status at 1st follow-up, were examined.

Four factors, patients’ age, comorbidity, presence of complications and certain symptoms (malaise, and gait uncertainty) were shown to be independent risk factors for severe neurological status.

Patients over 65 years were shown to have ORs of 9.88 and 4.91 based on their mRS and WNV-N index scores, respectively, while patients with 2 or more comorbidities had approximately 5-time- higher odds (5.2 and 4.59) for developing severe neurological status after hospital admission. „Weakness/malaise”, as a general symptom, was also a risk factor for severe neurological status following admission according to the mRS score (OR: 3.45) and „gait uncertainty” also constituted a risk (OR: 3.75) for worse neurological status, based on the WNV-N Index. Occurrence of complications significantly increased the odds of having a severe neurological status at 1st follow-up. The OR value was as high as 6.5, based on the WNV-N Index, while every patient who developed complications had a severe neurological status according to the mRS assessment. (Table 7).

**Table 7:** Risk factors for severe neurological status following hospital admission and/or at 1st follow-up, based on patients’ mRS scores (A) and WNV-N index scores (B)

Risk factors	OR	95% C.I.
**(A) mRS** (moderately severe/ severe neurological status)		
Age (< 65 years / 65+ years) (*N* = 66)	9.88	2.846–34.299
Comorbidities (2 or more) (no / yes) (*N* = 66)	5.20	1.577–17.149
Weakness/malaise (no / yes) (*N* = 66)	3.45	1.120–10.670
Complications (no / yes) (*N* = 43)	--^a^	
**(B) WNV-N Index** (moderately severe/ severe neurological status)		
Complications (no / yes) (*N* = 43)	6.50	1.130–37.200
Age (< 65 years / 65+ years) (*N* = 66)	4.91	1.628–14.817
Comorbidities (2 or more) (no / yes) (*N* = 66)	4.59	1.530–13.778
Gait uncertainty (no / yes) (*N* = 66)	3.75	1.126–11.123

^a^ All patients with complications belonged to the „severe neurological status” category

The relationship between the neurological symptom interval, the time (in days) between the onset of neurological symptoms and hospital admission, as a continuous variable and the severity of the neurological status following hospital admission, were analyzed. Our results showed, that time was a protective factor: the shorter the neurological symptom interval, the greater the chances for patients to avoid severe neurological symptoms.

Each day’s increase in the neurological symptom interval significantly increased the risk for having a severe neurological status following hospital admission (0.799-fold and 0.688-fold, according to the WNV-N Index and mRS, respectively) (Table 8). Other associations between demographic, clinical, diagnostic and therapeutic characteristics and neurological status severity were analyzed, but results were non-significant (data not shown).

**Table 8:** The relationship between the time between the onset of neurological symptoms and hospital admission and the severity of the neurological status following hospital admission based on patients’ mRS scores (A) and WNV-N Index scores (B)

Based on mRS	B	S.E.	*p*-value	Exp (B)	95% C.I.
(A)					
Time between the onset of neurological symptoms and hospital admission (days)	−0.374	0.133	0.005	**0.688**	0.530–0.894
Based on WNV-N Index	B	S.E.	*p*-value	Exp (B)	95% C.I.
(B)					
Time between the onset of neurological symptoms and hospital admission (days)	−0.224	0.089	0.011	**0.799**	0.672–0.951

*R*^*2*^ = 0.310.

*R*^*2*^ = 0.211.

## Discussion

Our study provided detailed data on the demographic and clinical characteristics of WNV infected patients from four regional medical centers in Hungary. The age and gender distribution as well as the presence of comorbidities was comparable with previous studies [26]. Symptoms of WNV infection often include fever, headache, malaise, muscle pain, chills, vomiting, eye pain, rash, and fatigue [6]. In our study population, fever, weakness/malaise and nausea/vomiting were the most common general symptoms.

Serological testing was carried out in all cases and was positive for either IgM or IgM and IgG in all of the patients’ serum or CSF samples. However, PCR testing was performed in only 84% of the cases and gave positive results in only 25.8% of all cases. The low proportion of PCR positivity can be explained by the short-term viremia and low viral load in humans. The length of elapsed time between symptom onset and date of sampling, and the storage and shipment conditions might also have an impact on the viral RNA stability in clinical specimens [27]. Lineage 1 and lineage 2 WNV, have been known as causative agents of human disease [28] and lineage 2 WNV was initially identified in Hungary outside of sub-Saharan Africa [29]. Lineage 2 WNV infections were identified in all of our PCR-positive cases, suggesting lineage 2 WNV circulation in Hungary, in accordance with sequence data [27]. Although, direct sequence data are not available from our PCR-negative cases, exposure to WNV lineage 2 infection was the most probable in their cases as well.

Treatment for WNND mostly includes supportive therapy, including administration of pain medication and the treatment of secondary infections, while treatment of seizures or respiratory insufficiency may even require intensive care [6, 8]. In our study population, administration of mannitol and steroid was the most common (19.7%) relevant medication, while analgesics and antibiotic or combination of antiviral (empiric acyclovir) and antibiotic therapy were given in the majority of the cases.

Meningitis, as WNND, can be typified by onset of fever, headache, meningeal signs and photophobia, while, encephalitis can manifest itself as a mild confusional state or as severe encephalopathy and coma [6]. Paralysis in WWND is usually caused by damage to the anterior horn cells of the spinal cord [6]. Although enrollment criteria in our study included all patients with confirmed WNV infection, the vast majority of our patients developed some form of WNND, that is, meningitis, encephalitis, meningoencephalitis or acute flaccid but all of the patients had some type of neurological symptom. The high proportion of WNND patients was unsurprising, since the symptoms of WNV infections are often mild and atypical, therefore patients often only present at a hospital with more severe symptoms, which are typical for neuroinvasive disease [2]. Almost a third of the patients suffered from muscle weakness after hospital admission, which in most cases was mild to moderate but reflex and sensory involvement was comparatively uncommon. Other frequent neurological symptoms were headache, vertigo and confusion following hospital admission which are in line with previous studies [2].

A few studies have focused on analyzing the neurological outcomes of WNV-infected patients [8, 9]. According to one investigation, long-term neurological abnormalities were most frequently found in WNND patients with primary WNV encephalitis, while another study reported that younger age was the only significant predictor for neurological recovery [8, 9]. Investigations based on previous post-stroke studies have shown that motor function improved rapidly in the first 6–8 weeks following neural injury and reached a plateau around 3 months [30]. Therefore, we believed it reasonable to follow-up our patients - whose motor function had been impaired in the majority of the cases - within a 2–6 month interval. The occurrence of muscle weakness has been reported to be typical of WNV-associated acute flaccid paresis and is thought to be due to a poliomyelitis-like process, a pure motor impairment [31–33]. As expected, the proportion of neurological symptoms in our study population and patients’ status severity decreased by the 1st and 2nd follow-up intervals.

In a number of studies, advanced age, immunosuppression, hypertension, diabetes mellitus, cerebrovascular disease and other comorbidities have been shown to be associated with neuroinvasive infection and long-term sequelae [2, 10, 34, 35]. Accordingly, we found, that age above 65 years and having 2 or more comorbidities significantly increased the odds of severe neurological status after hospital admission. Among the presenting symptoms, “weakness/malaise” and “uncertain gait” were associated with higher ORs for severe neurological status. Although the reason for this finding is unclear, we hypothesize that “uncertain gait” as a symptom may imply consequent muscle weakness or vertigo and “weakness/malaise” may be a forerunner of neuroinvasive disease. A recent study showed that admission to the intensive care unit (ICU) predicted longer hospital stay, in-hospital death and survival with inability to walk independently in WNND patients [7]. Furthermore, 81% of these patients admitted to the ICU developed some kind of complication [7]. Accordingly, in our study, the occurrence of complications significantly increased the odds of patients having a severe neurological status at 1st follow-up, underlining the importance of supportive care in WNND in order to avoid complications.

Only a few studies have reported the number of days between the onset of neurological symptoms and hospital admission [36], however, to our knowledge, none have investigated their association with neurological symptom severity. Our most prominent finding, therefore, was that the time between the onset of symptoms and hospital admission influenced the severity of the neurological status in WNV-infected patients and shorter time intervals served as a protective factor. The longer the interval, the greater patients’ chances were for having severe neurological status after hospital admission.

Our findings regarding the above-described risk factors have several implications. Since elapsed time between neurological symptom onset and hospital admission affects symptom severity, it would be vital that patients with neurological symptoms present at their health care provider in time. Clinicians vat emergency departments are often the first to meet patients with a suspicion of WNV-infection. Thus, they need to be prepared to make timely care-related decisions when their patients present with various neurological symptoms, which often cause differential diagnostic difficulties due to the varied manifestations of WNV infection [5]. As reported previously, WNV infection can be an occupational hazard, affecting the younger, active population [37, 38], and a recent investigation found that younger patients were more at risk of developing critical WNND since age below 60 years predicted ICU admission [7]. In our study, two-thirds of the patients were below 65 years, indicating that the young, active population, particularly with comorbidities, was also a high-risk population. Thus, epidemiologic preventive measures, such as the prevention of mosquito bites and public education about the symptoms of WNV infections, among the general population appears important [7, 39].

Assessment of risks and the use of evaluation scales help clinicians make timely decisions regarding diagnostic and therapeutic interventions, especially in emergency settings where optimal allocation of resources is vital. The modified Rankin scale has been used for the assessment of functional assessment after stroke and meningitis [40] and also in WNND patients in a previous study [7], therefore we considered it convenient for assessing neurological status severity in our sample of patients. In comparison to the mRS, our scoring system, the WNV-N Index, took into account neurological symptoms typical for WNV neuroinvasive disease. Throughout the investigation, the two scores moved parallel with each other. Based on our observations, the use of assessment tools should be considered for patients presenting with neurological symptoms indicating neuroinvasive disease. However, further investigations are warranted for the development of the optimal WNV neurological assessment tool.

### Limitations

There are limitations to our study. Our investigation was conducted on a small group of patients. It was done retrospectively, and the neurological assessment was not sufficiently detailed regarding neuropsychological deficits. Also, the use of the mRS and WNV-N index in WNV-infected patients has not been validated.

## Conclusions

Our study was conducted on a sample of WNV-infected Hungarian patients with neurological symptoms, in a country among a few in Europe, where the incidence and prevalence of WNV infections has significantly increased over the past years.

Age, comorbidities, and complications during hospital stay significantly and independently increased the risks for severe neurological status in patients, during their hospital stay or short-term follow-up. We also identified a novel risk factor, longer time between onset of neurological symptoms and hospital admission, for the occurrence of severe neurological symptoms in patients.

Our findings imply that awareness regarding WNV infections and related neurological symptoms should be raised among the general population in WNV-affected areas. Clinicians need to be aware of their patients’ risk factors when the suspicion of a WNV-infection arises. Furthermore, the development of an optimal tool for assessing neurological status in WNV infection could be an important step for improving outcome for WNND patients.

## Data Availability

The data involved in the current study are available upon request. Anyone who is interested in the information should contact the corresponding author.

## Abbreviations

WNV: West Nile virus
WNND: West Nile neuroinvasive disease
AFP: Acute flaccid paresis
WNV-N Index: West Nile virus neurological index
IgA, IgG, IgM: Immunoglobulin A, G, M
RNA: Ribonucleic acid
RT-PCR: Reverse transcriptase-polymerase chain reaction
USUV: Usutu virus
NIS: Neuropathy impairment score
mRS: Modified rankin scale
OR: Odds ratio
CSF: Cerebrospinal fluid
CT: Computed tomography
MRI: Magnetic resonance imaging
GCS: Glasgow coma scale
PCR: Polymerase chain reaction
ICU: Intensive care unit
